# Genotype by Environment Interaction for Agro‐Morphological Traits and Grain Yield Stability in Barley ( *Hordeum vulgare* L.) Genotypes Using AMMI Analysis Under Acidic Soil Conditions in Ethiopia

**DOI:** 10.1002/pei3.70192

**Published:** 2026-07-24

**Authors:** Chigign Mengiste, Walelign Worku, Berhanu Abate, Alemayehu Kiflu, Gezahagn Kebede

**Affiliations:** ^1^ Department of Plant Sciences Wolkite University Wolkite Ethiopia; ^2^ School of Plant and Horticulture Sciences Hawassa University Hawassa Ethiopia; ^3^ Holetta Agricultural Research Center (HARC) Holetta Ethiopia

**Keywords:** AMMI analysis, barley genotypes, genotype by environment interaction, grain yield, lime, soil acidity, yield stability

## Abstract

Barley production in the Ethiopian highlands is constrained by soil acidity and the lack of acid‐tolerant genotypes. This study evaluated the effects of environment, genotype, and their interaction on barley performance and identified suitable genotypes for acidic soils. Ten barley genotypes, including eight accessions, one local variety, and one released cultivar, were tested under acidic and lime‐treated soil conditions using a randomized complete block design. The results on the additive main effects and multiplicative interactions (AMMI) analysis showed that environment explained 60.45% of the total variation, genotype 32.38%, and genotype by environment interaction accounted for 7.18%. The first and the second interaction principal component axes were significant and cumulatively accounted for 80.59% of the total interaction variance. Analysis of the first two principal components showed that E7 and E8 in the Gumer district, representing fully limed and unlimed soils, respectively, contributed most to the genotype by environment interaction. In contrast, E5 and E6 in the Hulla district, representing unlimed and fully limed soils, respectively, contributed least, while E1–E4 had moderate effects. Grain yields in limed environments consistently exceeded the grand mean of 2.79 t ha^−1^, whereas the unlimed environments produced substantially lower yields across. The first two AMMI components identified genotypes G1, G3, G9, and G10 as superior performers, combining high yield with stability. Therefore, expanding the cultivation of improved variety, G9, and further evaluating genotype G1, G3 and G10, and lime application could improve barley production and food security in the southern highlands of Ethiopia.

## 1 Introduction

Barley holds significant economic importance globally, contributing to agriculture, the food and beverage industry, and livestock feed production. According to the Food and Agriculture Organization (FAO), barley ranks among the world's leading cereal crops in terms of production (Giraldo et al. 2019). In Ethiopia, it is the fifth most important crop next to teff ( *Eragrostis tef* ), maize ( *Zea mays* L.), sorghum ( *Sorghum bicolor* L.), and wheat ( *Triticum aestivum* L.) (Bizuneh and Abebe 2019). Barley shares 9.3% of the total cereal area and 7.7% of the total cereal production. Ethiopia leads Africa in barley production, producing 2.1–2.5 million tons annually from approximately 1 million hectares, with an average grain yield of 1.9–2.2 t ha^−1^ (Ahmed et al. 2024). While the area under barley, total production, and yield ha^−1^ have increased by 0.57%, 2.19%, and 1.62% year^−1^, respectively, these growth rates remain slow compared to other major cereals (Diriba 2020). Barley is primarily cultivated by smallholder farmers in Ethiopia's highland regions (Rapsomanikis 2015), who also cultivate 95% of the main crops of the country (Aweke 2017). Low‐yielding barley varieties (Mulatu and Grando 2006), low soil pH, and related soil infertility are among the primary challenges confronting barley production in the Ethiopian highlands (Desalegn et al. 2017).

Acidic soils cover about 30% of the Earth's land surface and more than 50% of potentially arable land, particularly in tropical and subtropical regions (Kochian et al. 2015). In Ethiopia, the problem is most severe in high‐rainfall highland areas, where approximately 41% of arable land is affected by acidity‐related infertility (Mesfin 2007; Taye 2007). Of this, 27.7% is moderately acidic (pH 4.5–5.5) and 13.2% is strongly acidic (pH < 4.5) (Taye 2007). High rainfall accelerates the leaching of base cations, contributing to phosphorus deficiency (Chimdi et al. 2012; Melese and Gebrekidan 2014). In the north‐central and southwestern highlands, about 80% of Nitisols and Luvisols are strongly acidic (Eyasu 2016). Similar conditions in southern Ethiopia also limit barley production and threaten the food and economic security of local farmers (Fekadu et al. 2019). Consequently, soil acidity remains a major barrier to agricultural development in the Ethiopian highlands (Tamene et al. 2017). Improving crop production on such acidic, phosphorus‐deficient soils requires integrated management approaches, including liming, inorganic fertilizers, organic amendments, and the use of aluminum‐tolerant genotypes (Evans et al. 2013). Among the management options, liming is the most effective method for raising soil pH, improving soil properties, and enhancing microbial activity (Paradelo et al. 2015), which promotes organic matter decomposition (Grover et al. 2017). These effects can increase the availability of essential plant nutrients (Andersson and Nilsson 2001), thereby improving crop growth and yield.

Genetic variation in aluminum toxicity tolerance among plant species and genotypes also provides a valuable strategy for addressing soil fertility challenges in acidic soils (Mesdag and Balkema‐Boomstra 1984; Foy 1988). However, because the performance of genotypes can differ across environments, it is critical to evaluate them under diverse conditions to accurately determine their genetic potential and adaptability (Bilgin et al. 2018). The concept of genotype‐by‐environment interaction (GEI) pertains to the varying performance or ranking of genotypes in different environments, which significantly influences the selection and recommendation of cultivars best suited for specific growing conditions (Gauch Jr 2006; Mohammadı et al. 2012). GEI is vital in assessing genotype stability and adaptability, as all yield‐related traits are influenced by both genetic and environmental factors (Moldovan et al. 2000; Doehlert et al. 2001; Annicchiarico 2002). Conducting stability analyses is essential for identifying high‐yielding and consistent genotypes across a range of environments and growing seasons (Yan and Hunt 2001; Sharma et al. 2010; Verma et al. 2016). Consequently, multi‐environment trials (METs) are effectively employed to evaluate genotypes in varied environmental conditions (Bavandpori et al. 2015). Genotype performance may differ under various acidic and lime‐treated soil conditions due to GEI effects that are influenced by soil characteristics, climatic conditions, and biotic factors (Yan and Kang 2002; Jeberson et al. 2017).

The analysis of variance (ANOVA) partitions the total sum of squares into components attributable to environment, genotype, and GEI, but it does not further decompose the interaction component (Nadeem and Islam 2007). In addition, the use of the genotype plus genotype by environment (GGE) biplot emphasizes the genotype and genotype by environment (GE) interaction effects, making it effective for mega‐environment analysis and genotype evaluation, including attribute‐based genotype ranking (Yan et al. 2007). However, its display of a single “ideal cultivar” may penalize increases in a genotype's main effect, overlook potential yield gains arising from GE interactions, and depend on specific dataset properties for the validity of its average‐environment coordinate (AEC) or the average tester coordinate (ATC) ordinate (AEC/ATC) axis (Gauch Jr 2006). In general, the GGE approach explicitly recognizes genotype (G) and genotype by environment (GE) interaction effects as integral components of cultivar evaluation and plant breeding (Yan and Kang 2002). In contrast, the AMMI model identifies multiple potential winners across one or more mega‐environments, as well as universal winners. This diversity provides plant breeders and agronomists with greater flexibility and enhances the likelihood of developing new successful cultivars. Additionally, the AMMI display is robust and does not rely on specific dataset properties (Gauch Jr 2006). The AMMI model is an extension of ANOVA and is widely used for the analysis of multi‐environment varietal trials (Gauch 1992). Specifically, AMMI separates the additive effects, which are captured by ANOVA and represent the main effects of genotypes and environments, from the multiplicative effects, which account for GEI and are modeled by principal component analysis (PCA). It considers the main effect of a genotype's average yield across environments as additive, while the specific way that genotype responds differently to various environments is reflected in the multiplicative effect. Therefore, the AMMI model combines ANOVA and PCA to partition both the additive and multiplicative components of the GEI, providing a clearer understanding of genotype performance across environments (Gauch 2007; Mukherjee et al. 2013). By applying PCA to the interaction component, AMMI identifies the major patterns underlying GEI, while AMMI biplots facilitate interpretation through graphical visualization of genotype‐environment relationships (Thillainathan and Fernandez 2001).

Although the AMMI model has been widely applied in various crops in Ethiopia (Etana and Merga 2021), its use in evaluating barley genotypes under acidic and lime‐treated soil environments in the southern highlands of Ethiopia remains limited. Although Ethiopia is recognized as a center of barley diversity, yields remain low for many farmers due to genetic, environmental, and socioeconomic constraints (Central Statistics Agency (CSA) 2017). In addition to the study on the soil fertility benefits of liming, based on the genotype responses, evaluating barley genotypes for grain yield and yield stability offers a promising strategy for mitigating the adverse effects of aluminum and manganese toxicity in acidic soils. Therefore, this study was conducted to evaluate the effects of genotype, environment, and GEI on grain yield and yield stability under lime‐amended and acidic soil conditions using the AMMI model, as well as on other agronomic and phenological traits in the southern highlands of Ethiopia. The study also aimed to identify superior barley genotypes that could broaden the seed sources available in the study districts.

## 2 Materials and Methods

### 2.1 Description of the Study Sites

The experiment was conducted in eight different environments in the southern highlands of Ethiopia during the main cropping seasons of 2021 and 2022. Environments E1, E2, E5, and E6 were located in the Hulla district, which is situated at 6°31′22.77″N, 38°31′3.64″E, with an altitude of 2675 m a.s.l. The district's long‐term mean annual rainfall is 1327 mm, with mean minimum and maximum temperatures of 6.9°C and 18.8°C, respectively. Environments E3, E4, E7, and E8 were located in the Gumer district, which is situated at 8°1′0.62″N, 38°6′38.48″E, at an altitude of 2967 m a.s.l., with a long‐term average rainfall of 1300 mm and mean minimum and maximum temperatures of 7.8°C and 19.7°C, respectively. All sites represent major barley‐growing environments in the southern region of the country, where soil acidity is the most limiting production factor, as is the case in most highland areas of Ethiopia. The geographic locations, climates, and initial soil characteristics are presented in Table 1.

**TABLE 1:** Geographic location, climate condition and initial soil characteristics of the test environments.

Parameters	Environment							
	E1	E2	E3	E4	E5	E6	E7	E8
Altitude (m a.s.l.)	2675	2675	2967	2967	2675	2675	2967	2967
Latitude (N)	6°31′22.77″	6°31′22.77″	8°1′0.62″	8°1′0.62″	6°31′22.77″	6°31′22.77″	8°1′0.62″	8°1′0.62″
Longitude (E)	38°31′3.64″	38°31′3.64″	38°6′38.48″	38°6′38.48″	38°31′3.64″	38°31′ 3.64″	38°6′38.48″	38°6′38.48″
ARF (mm)	1327	1327	1300	1300	1327	1327	1300	1300
Minimum temp (°C)	6.4	6.4	7.8	7.8	6.4	6.4	7.8	7.8
Maximum temp (°C)	19	19	19.7	19.7	19	19	19.7	19.7
Textural class	Clay loam	Clay loam	Clay loam	Clay loam	Clay loam	Clay loam	Clay loam	Clay loam
BD (g cm^−3^)	1.39	1.39	1.44	1.44	1.39	1.39	1.44	1.44
Sand (%)	30	30	36	36	30	30	36	36
Clay (%)	30	30	30	30	30	30	30	30
Silt (%)	40	40	34	34	40	40	34	34
pH (H_2_O)	4.58	4.58	4.30	4.30	4.58	4.58	4.30	4.30
TN (%)	0.33	0.33	0.31	0.31	0.33	0.33	0.31	0.31
AP (mg kg^−1^)	9.84	9.84	8.71	8.71	9.84	9.84	8.71	8.71
Calcium (Ca^2+^) (mg kg^−1^)	2054.62	2054.62	1103.77	1103.77	2054.62	2054.62	1103.77	1103.77
CEC (mEq/100 g)	32.44	32.44	28.38	28.38	32.44	32.44	28.38	28.38
Exchangeable acidity (EA) meq/100 g soil	2.91	2.91	3.27	3.27	1.64	1.64	3.27	2.27
Lime^a^	Not applied	Applied	Not applied	Applied	Not applied	Applied	Not applied	Applied

^a^ Lime was applied based on soil exchangeable acidity levels at 70% of the calculated rate for E2, E4, and at 100% estimated rate for E6 and E8; E1 = Hulla, unlimed soil in 2021; E2 = Hulla, Limed soil, 2021, E3 = Gumer, Unlimed soil, 2021; E4 = Gumer, Limed soil, 2021; E5 = Hulla, Unlimed soil, 2022; E6 = Hulla, Limed soil, 2022, E7 = Gumer, Unlimed soil, 2022; E8 = Gumer, Limed soil, 2022; Hulla = Hulla district, Gumer = Gumer district.

These environments were identified as E1 (Hulla district, unlimed soil, 2021), E2 (Hulla district, limed soil, 2021), E3 (Gumer district, unlimed soil, 2021), E4 (Gumer district, limed soil, 2021), E5 (Hulla district, unlimed soil, 2022), E6 (2022 Hulla district, limed soil, 2022), E7 (Gumer district, unlimed soil, 2022), E8 (Gumer district, limed soil, 2022). Environment E2 and E4 received 70% of the recommended lime rate (2.12 and 4.97 t ha^−1^, respectively), while environments E6 and E8 received 100% of the recommended lime (2.12 and 4.97 t ha^−1^, respectively), with all rates determined based on each site's exchangeable acidity levels. Hulla and Gumer districts are among the major barley growing areas and were generally characterized by a mixture of highland crops and livestock production system. Barley was the most dominated cereal crop in household food security and cash income.

### 2.2 Experimental Materials

Ten food barley genotypes, including eight accessions, one local control, and one released cultivar (Table 2), were used in the study. The eight accessions were selected from an initial screening of 200 barley materials sourced from the Ethiopian Biodiversity Institute (EBI). The selection of the eight genotype/accession was primarily done based on better grain yield performance observed during the preliminary evaluation. The local variety, *Juma Tikur*, is widely grown in the highlands of the Gumer district in southern Ethiopia. *Juma Tikur* was one of a few barley landraces relatively adapted to acid‐prone soils of the area, helping sustain local livelihoods where many varieties fail because of severe soil acidity and related fertility problems. The seed of the check cultivar (HB‐1307), which is a six‐row cultivar released in 2006 for the mid and highlands of Ethiopia, was obtained from Holleta Agriculture Research Center (HARC), Ethiopia. Despite its recommendation for the mid and highlands, the cultivar HB‐1307 was not widely adopted by farmers.

**TABLE 2:** Description of the 10 experimental barley genotypes and their sources.

No.	Genotypes	Designation	Remark	Source	Maturity (days)^b^
1	217176b	G1	Accession	EBI	107–129 (118.5)
2	234911	G2	Accession	EBI	110–133 (122.6)
3	240478a (white)	G3	Accession	EBI	104–128 (115.9)
4	240478b (black)	G4	Accession	EBI	100–124 (112.4)
5	215453b	G5	Accession	EBI	110–133 (121.6)
6	215454a	G6	Accession	EBI	100–128 (115.6)
7	27895a	G7	Accession	EBI	109–130 (120.0)
8	208855b	G8	Accession	EBI	111–134 (122.5)
9	HB‐1307	G9	Released cultivar	HARC	132–148 (140.0)
10	Juma Tikur	G10	Local control	Gumer^a^	128–151 (139.7)

Abbreviations: EBI = Ethiopian Biodiversity Institute, HARC = Holleta Agricultural Research Centre (Ethiopia).

^a^ Farmers from Gumer district.

^b^ Based on the 2021 data and the parenthesis are mean values.

### 2.3 Determination of Lime Requirements

The amount of lime for each site was calculated using the exchangeable acidity method, which was done on the mass of soil per 0.15 m hectare‐furrow‐slice, bulk density, and exchangeable acidity, assuming that one mole of exchangeable acidity would be neutralized by an equivalent mole of CaCO_3_ (Agegnehu et al. 2019).

$$\mathrm{LR}=\frac{\left(\text{cmolEA}\;{\mathrm{kg}}^{-1}\text{of soil}\right)\times 0.15\;m\times 10^{4}\;m^{2}\times \mathrm{BD}\left(\mathrm{mg}\;m^{-3}\right)\times 1000\;}{2000}$$

where LR = lime rate (kg ha^−1^); EA = exchangeable acidity; B.D = bulk density of soil.

A limestone with 89.98% quality was used to calculate the required amount of lime for each site.

### 2.4 Liming, Experimental Design and Agronomic Management

After 10 composite soil samples were collected using the zigzag method, the total area was divided into two divisions, one with lime applied and the other as an unlimed farm area. The calculated lime rates were applied 1 month before planting to allow adequate time for soil reaction. Environments E2 and E4 received 70% of the recommended lime requirement to evaluate a more economical and farmer‐relevant liming strategy, considering the cost and limited accessibility of lime in the area. This treatment also enabled the assessment of genotype responses under partial soil acidity amelioration. In contrast, environments E6 and E8 received the full‐recommended lime rate, which was calculated based on the exchangeable acidity levels of the soils in each site. A randomized complete block design (RCBD) replicated three times was used for the evaluation of barley genotypes, in both the limed and unlimed soils, independently. The genotypes were then assigned randomly within a block of each soil environment. Sowing was conducted on varied dates, depending on the onset of rain at each location and in each year. In 2021, sowings were done on August 5 and 12 for Gumer and Hulla, and in 2022, they were on August 4 and 14, respectively. Each genotype was sown on a plot area of 2.1 m^2^ consisting of six rows of 1.75 m length and with 0.2 m between rows. The spacing between plots and blocks was 0.5 and 1 m, respectively. One border row on both sides of the plot was left as a guard plant to avoid the border effects for each plot. Thus, the four middle rows with a net plot size of 1.75 m × 0.8 m (1.4 m^2^) were used for data collection. The recommended seed rate of 100 kg ha^−1^ was used at sowing for each genotype. The recommended fertilizer at a rate of 100 kg ha^−1^ of TSP and 100 kg ha^−1^ of urea was used. The full rate of TSP and half of the urea was applied at sowing and the remaining urea was applied at tillering uniformly to all experimental plots. Two hand weedings and one herbicide application were done at all testing environments to control weed competition. All other crop management practices were uniformly applied to all environments whenever required.

### 2.5 Data Collection

Plant height and number of leaves plant^−1^ were assessed for each of 10 randomly selected plants from the middle four rows at physiological maturity and flower initiation, respectively. The main plant was tagged early, before tillering, and the number of productive tillers contributing to grain yield was counted within a 1 m^2^ area for each plot. The aboveground biological yield was measured at physiological maturity after drying to a constant weight. Data on barley grain yield and yield components were collected at harvest from the middle four rows (1.4 m^2^). Manual harvesting and threshing were done on a plot basis, and the grain moisture content was adjusted to 12.5%. The weight of 1000 seeds was measured using a sensitive balance. The spike length and the number of seeds spike^−1^ were measured from 10 randomly selected plants for each plot at harvest. The number of days from sowing to 50% flowering and 50% physiological maturity were recorded for each plot.

### 2.6 Data Analysis

#### 2.6.1 Analysis of Variance

The analysis of variance (ANOVA) procedures of the statistical analysis system (SAS) general linear model (GLM) version 9.4 was used for individual environments and combined across environments (SAS (Statistical Analysis System) 2012). To determine the validity of each environment analysis, Bartlett's test for homogeneity of variance was done by the SAS computer packages prior to computing ANOVA. Moreover, the homogeneity of error variance for combined ANOVA was tested using the F‐max method of Hartley (1950), which is based on the ratio of the larger mean square error (MSE) from the separate analysis of variance to the smaller MSE (Table 3). The genotype was considered fixed and the environment was considered random, therefore a linear mixed model was used for data analysis. The genotype was tested against the GEI mean square while the environment and the interaction effects were tested against the replication mean square and residuals, respectively. The mean comparison was done using Duncan's multiple range test at a 5% probability level following the procedures suggested by Gomez and Gomez (1984). All data used in this study are provided in the Supporting Information.

**TABLE 3:** Homogeneity of error variance estimation using Hartley test for measured traits of barley genotypes grown under diverse environments.

Traits	Mean square error								Larger	Smaller	Hartley test
	E1	E2	E3	E4	E5	E6	E7	E8			
GY	0.0135	0.0183	0.0330	0.0274	0.0160	0.0397	0.0256	0.0387	0.0397	0.0135	2.9
ABY	0.1871	0.1866	0.4927	0.3372	0.2687	0.2769	0.1631	0.2797	0.4927	0.1631	3.0
HI	0.0009	0.0010	0.0004	0.0004	0.0004	0.0009	0.0007	0.0005	0.0010	0.0004	2.5
SL	0.1229	0.1620	0.1589	0.2505	0.2462	0.3588	0.1900	0.1879	0.3588	0.1229	2.9
NSPS	5.5192	3.6607	5.9993	5.0233	3.0427	3.5366	5.9402	3.0439	5.9993	3.0427	2.0
PH	1.3768	1.3519	1.2618	1.2242	0.6988	1.0464	1.2925	1.6708	1.6708	0.6988	2.4
NL	0.2270	0.1909	0.4129	0.2458	0.1495	0.1551	0.2606	0.1656	0.4129	0.1495	2.8
PT	0.0400	0.0786	0.0914	0.0495	0.0403	0.0368	0.0910	0.0479	0.0914	0.0368	2.5
TSW	4.3902	3.9177	2.5471	5.6073	2.4369	4.3628	5.5705	4.6803	5.6073	2.4369	2.3
DF	1.9296	1.3370	3.2185	2.8037	3.7148	1.4296	1.9519	1.4704	3.7148	1.3370	2.8
DM	5.4185	5.0481	3.9074	3.4333	3.3667	3.7185	3.7519	3.4333	5.4185	3.3667	1.6

Abbreviations: ABY = Aboveground biological yield (t ha^−1^), DF = Days to 50% flowering, DM = Days to 50% maturity, E1 = Hulla, Unlimed soil in 2021, E2 = Hulla, limed soil (2021), E3 = Gumer, Unlimed soil (2021), E4 = Gumer, Limed soil (2021), E5 = Hulla, Unlimed soil (2022), E6 = Hulla, Limed soil (2022), E7 = Gumer, Unlimed soil (2022), E8 = Gumer, Limed soil (2022), GY = Grain yield (t ha^−1^), HI = Harvest index, NL = Number of Leaves plant^−1^, NSPS = Number of seeds spike^−1^, PH = Plant height (cm), PT = Productive tiller m^−2^, SL = Spike length (cm), TSW = 1000 seed weight (g), means followed by a common superscript letter within a row are not significantly different from each other at P < 0.05.

#### 2.6.2 AMMI Analysis

Additive main effects and multiplicative interactions (AMMI) is one of the most widely used models to explain the GEI of a multi‐environment trial and categorize the genotypes into narrow or wider adaptation (Crossa et al. 1990). The statistical analysis of the AMMI model was tested using Gollob's F‐test procedure (Gollob 1968). The AMMI analysis was performed using the Genotype by Environment Analysis with R (GEA‐R) version 4.0 (Pacheco‐Gil et al. 2015).

## 3 Results

### 3.1 Analysis of Variance

**FIGURE 1:**
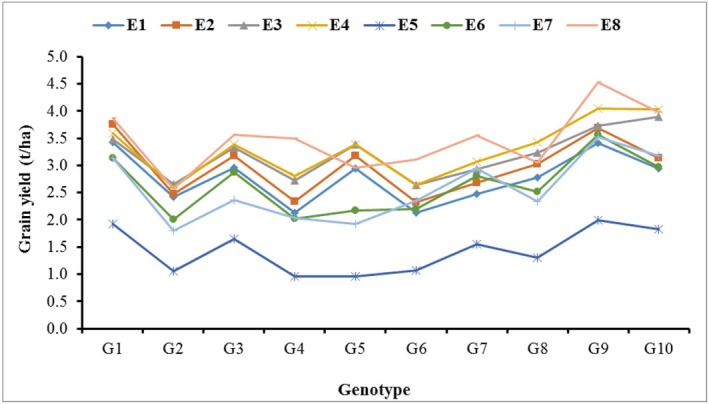
Genotype by environment interaction (crossover) for grain yield of 10 barley genotypes grown at eight environments. G1 = 217176b, G2 = 234911, G3 = 240478a, G4 = 240478b, G5 = 215453b, G6 = 215454a, G7 = 27895a, G8 = 208855b, G9 = HB‐1307 (released variety), G10 = *Juma Tikur* (local variety), E1 = Hulla, unlimed soil in 2021, E2 = Hulla, limed soil (2021), E3 = Gumer, unlimed soil (2021), E4 = Gumer, limed soil (2021), E5 = Hulla, unlimed soil (2022), E6 = Hulla, limed soil (2022), E7 = Gumer, unlimed soil (2022), E8 = Gumer, limed soil (2022).

The combined analysis of variance revealed that the main effects of environments (E), genotypes (G), and GEI were highly significant (*p* < 0.001) for agronomic and phenology traits across the test environments (Table 4). The GEI effects also showed a crossover interaction pattern for grain yield in all environments except E5. In E5, there was no genotype that exhibited interaction or change in rank for grain yield across the environments (Figure 1).

**TABLE 4:** Combined analysis of variance for quantitative traits of 10 barley genotypes tested in eight environments under soil acidic conditions.

Traits	Mean squares					CV	*R* ^2^
	Env. (E) (df = 7)	Rep (Env.) (df = 16)	Geno (G) (df = 9)	GxE (GEI) (df = 63)	Error (df = 144)		
GY	12.24***	0.04^NS^	5.10***	0.16***	0.03	5.84	0.97
ABY	65.96***	0.37^NS^	16.24***	1.20***	0.27	7.20	0.95
HI	0.02***	0.00^NS^	0.02***	0.00***	0.00	6.66	0.82
SL	29.01***	0.14^NS^	19.95***	1.13***	0.21	6.46	0.94
NSPS	630.65***	4.15^NS^	2339.12***	33.39***	4.47	7.03	0.98
PH	5151.03***	1.50^NS^	521.86***	80.16***	1.24	1.20	0.99
NL	184.34***	0.57**	12.96***	3.25***	0.23	10.52	0.98
PT	4.61***	0.08^NS^	3.98***	0.21***	0.06	9.78	0.91
TSW	226.84***	3.30^NS^	458.11***	12.07***	4.19	4.78	0.92
DF	331.45***	1.91^NS^	2735.37***	8.99***	2.23	2.35	0.99
DM	2546.90***	9.33**	2509.03***	25.08***	4.01	1.63	0.99

Abbreviations: ABY = aboveground biological yield (t ha^−1^), DF = days to 50% flowering (days), DM = days to 50% maturity (days), GY = grain yield (t ha^−1^), HI = Harvest index, NL = number of leaves plant^−1^, NSPS = number of seeds spike^−1^, PH = plant height (cm), PT = productive tiller m^−2^, SL = spike length (cm), TSW = 1000 seed weight (g), ∗∗ and ∗∗∗ indicate significance at the 0.01 and 0.001 probability levels, respectively, and NS = not‐significant.

### 3.2 Effect of Environments on Barley Performance

The analysis of variance (ANOVA) revealed that environmental conditions had highly significant effect (*p* < 0.001) on all measured agronomic and phenological traits (Table 5). Grain yield (GY), aboveground biological yield (ABY), number of seeds spike^−1^ (NSPS), and productive tillers (PT) reached their highest values at E8, which received the full lime rate. Markedly, NSPS at E8 was statistically similar to E3 and E4, while PT was also comparable to E4. Higher harvest index (HI) values were recorded at E1, E2, E3, and E4, meanwhile, plant height (PH), number of leaves plant^−1^ (NL), and thousand‐seed weight (TSW) were highest at E4. The longest durations to flowering and maturity were observed at E3 and E4. Overall, the Gumer environments (E3, E4, and E8) demonstrated superior performance for most traits, likely due to their lower initial soil acidity and the beneficial effects of lime application, particularly at E4 and E8. In contrast, environment E1, E2, E5, E6, and E7 generally showed lower performance, except for HI in E1 and E2. Within the Hulla environments, E5 exhibited very poor performance across most of the measured traits, while E7 also performed poorly except for HI, TSW, and days to maturity. Across the test sites, liming shortened the duration of phenological stages, likely through improvements in soil chemical properties and nutrient availability. Generally, the results indicated that the environmental factors, particularly soil acidity levels and lime application, significantly influenced barley growth, phenology, and grain yield performance in both districts (Table 5).

**TABLE 5:** Mean performance for quantitative traits of 10 barley genotypes evaluated in eight environments under acidic and lime‐treated soil conditions.

Traits	E1	E2	E3	E4	E5	E6	E7	E8	Mean	*p*
GY	2.76^d^	2.98^c^	3.20^b^	3.30^b^	1.43^f^	2.63^e^	2.56^e^	3.47^a^	2.79	< 0.0001
ABY	6.96^cd^	7.24^c^	8.19^b^	8.34^b^	4.46^e^	6.87^cd^	6.62^d^	9.46^a^	7.27	< 0.0001
HI	0.40^ab^	0.41^a^	0.39^ab^	0.40^ab^	0.32^c^	0.38^bc^	0.38^bc^	0.37^c^	0.38	< 0.0001
SL	6.55^d^	7.03^c^	7.65^b^	8.31^a^	5.02^e^	7.65^b^	7.11^c^	7.36^bc^	7.08	< 0.0001
NSPS	25.96^d^	28.61^c^	33.72^a^	34.66^a^	21.40^e^	32.04^b^	30.20^c^	33.91^a^	30.06	< 0.0001
PH	95.77^c^	94.66^d^	104.75^b^	108.95^a^	64.86^g^	92.14^e^	90.64^f^	92.14^e^	92.99	< 0.0001
NL	3.31^d^	4.29^c^	7.27^b^	9.41^a^	2.51^f^	3.13^de^	2.88^ef^	3.34^d^	4.52	< 0.0001
PT	2.28^e^	2.60^cd^	2.49^d^	3.01^a^	1.77^f^	2.73^bc^	2.24^e^	2.82^ab^	2.49	< 0.0001
TSW	40.59^c^	43.51^b^	43.95^b^	46.41^a^	37.35^d^	42.86^b^	43.87^b^	44.32^b^	42.86	< 0.0001
DF	61.87^ef^	60.70^fg^	69.30^a^	67.57^b^	64.53^c^	59.90^g^	63.10^d^	61.90^e^	63.61	< 0.0001
DM	113.07^f^	111.47^g^	134.37^a^	132.80^a^	118.60^d^	115.17^e^	129.60^b^	126.70^c^	122.72	< 0.0001

Abbreviations: ABY = Aboveground biological yield (t ha^−1^), DF = Days to 50% flowering, DM = Days to 50% maturity, E1 = Hulla, unlimed soil in 2021, E2 = Hulla, limed soil (2021), E3 = Gumer, unlimed soil (2021), E4 = Gumer, limed soil (2021), E5 = Hulla, unlimed soil (2022), E6 = Hulla, limed soil (2022), E7 = Gumer, unlimed soil (2022), E8 = Gumer, limed soil (2022), GY = Grain yield (t ha^−1^), HI = Harvest index, NL = Number of leaves plant^−1^, NSPS = Number of seeds spike^−1^, PH = Plant height (cm), PT = Productive tiller m^−2^, SL = Spike length (cm), TSW = 1000 seed weight (g), means followed by a common superscript letter within a row are not significantly different from each other at P < 0.05.

### 3.3 Mean Performance of Genotypes

Analysis of variance (ANOVA) showed highly significant (*p* < 0.001) genotypic differences for all growth and phenological traits (Table 6). Genotype G1 (acc. 217176b) produced the highest aboveground biomass yield (ABY), harvest index (HI), plant height (PH), and spike length (SL). For most traits, except the PH and SL, G1 was statistically comparable to G9 (HB‐1307) and G10 (*Juma Tikur*). Genotype G3 (acc. 240478a) also exhibited a high HI. The released variety G9 and the local variety G10 had the highest number of leaves plant^−1^ (NL), productive tillers (PT), and seeds spike^−1^ (NSPS), but produced shorter spikes than the other genotypes. Genotype G2 (acc. 234,911) recorded the highest thousand‐seed weight (TSW), statistically comparable to G3, despite its relatively poor performance for most other measured traits. In contrast, G4 (acc. 240478b) and G6 (acc. 215454a) produced the lowest ABY. The G9 and G10 exhibited the longest flowering and maturity periods. Among the collected accessions, G8 (acc. 208855b) showed the longest maturity duration, while G1 had intermediate flowering and maturity times.

**TABLE 6:** Mean performance for agronomic and phonologic traits of 10 barley genotypes grown in eight environments under acidic and lime‐treated soil conditions.

Genotype	ABY	PH	NL	PT	HI	SL	NSPS	TSW	DF	DM
G1	8.19^a^	103.07^a^	4.91^bc^	2.88^b^	0.40^ab^	8.31^a^	31.14^b^	41.37^de^	59.08^d^	117.71^d^
G2	6.58^c^	92.32^d^	3.59^g^	2.14^de^	0.33^e^	6.15^e^	24.79^de^	49.16^a^	60.92^c^	122.29^b^
G3	7.29^b^	95.39^b^	4.15^d‐f^	2.30^cd^	0.40^ab^	7.11^d^	23.82^e^	48.62^ab^	56.17^f^	114.75^e^
G4	5.90^d^	87.40^f^	3.83^fg^	1.96^e^	0.39^bc^	7.48^b‐d^	23.00e	43.67^c^	54.63^g^	111.75^f^
G5	7.12^b^	90.53^e^	4.51^cd^	2.34^cd^	0.36^d^	7.71^b^	25.81^cd^	46.92^b^	60.96^c^	121.33^bc^
G6	6.35^cd^	90.76^e^	4.06^ef^	2.26^cd^	0.36^d^	7.25^cd^	24.01^de^	40.32^e^	56.96^ef^	114.79^e^
G7	7.45^b^	95.04^bc^	4.56^cd^	2.39^c^	0.37^cd^	7.65^bc^	26.89^c^	39.58^e^	57.83^de^	120.08^c^
G8	7.31^b^	94.14^c^	4.29^de^	2.44^c^	0.37^cd^	7.66^bc^	24.61^de^	40.83^e^	62.92^b^	122.75^b^
G9	8.46^a^	94.39^bc^	6.11^a^	3.26^a^	0.42^a^	6.19^e^	48.14^a^	42.86^cd^	82.88^a^	141.50^a^
G10	8.02^a^	86.82^f^	5.18^b^	2.95^b^	0.41^ab^	5.33^f^	48.45^a^	35.23^f^	83.75^a^	140.25^a^
*p*	< 0.0001	< 0.0001	0.0005	< 0.0001	< 0.0001	< 0.0001	< 0.0001	< 0.0001	< 0.0001	< 0.0001

Abbreviations: ABY = Aboveground biological yield (t ha^−1^), DF = Days to 50% flowering, DM = Days to 50% maturity, G1 = 217176b, G10 = *Juma Tikur* (local variety), G2 = 234911, G3 = 240478a, G4 = 240478b, G5 = 215453b, G6 = 215454a, G7 = 27895a, G8 = 208855b, G9 = HB‐1307 (released variety), HI = Harvest index, NL = number of leaves plant^−1^, NSPS = number of seeds spike^−1^, PH = plant height (cm), PT = productive tiller m^−2^, SL = spike length (cm), TSW = 1000 seed weight (g), means followed by a common superscript letter within a colomn are not significantly different from each other at P < 0.05.

The ANOVA results demonstrated that genetic differences among the tested barley genotypes had a highly significant effect (*p* < 0.001) on grain yield (GY) performance across all testing environments (Table 7). Genotypes G9, G1, and G10 consistently achieved higher GY than the others. In particular, the released cultivar HB‐1307 (G9) exhibited superior GY across all environments, performing statistically on par with G1 (acc. 217176b), except in environment E8, where G1 yielded less than G9. The local variety *Juma Tikur* (G10) also performed grain yield comparable to G9 in most environments, except E1, E2, and E6. Genotype G3 (acc. 240478a) emerged as a promising candidate, ranking just below G1 in both individual environments and overall mean of GY. Accessions G5, G7, and G8 showed moderate performance, while G2, G4, and G6 recorded lower grain yields (Table 7). Overall, G9, G1, G10, and G3 exceeded the grand mean yield (2.79 t ha^−1^) across all the test environments except at E5, where none of the genotypes exceeded the grand mean. In E5, GY ranged from the lowest of 0.96 t ha^−1^ (G4 and G5) to the highest of 1.99 t ha^−1^ (G9). Across environments, the released variety (G9) produced the highest grain yield (4.53 t ha^−1^) at E8 and the second‐highest yield (4.05 t ha^−1^) at E4. Therefore, based on their superior agronomic and grain yield performance, HB‐1307, Juma Tikur, acc.217176b, and acc.240478a identified as promising genotypes. The released variety, HB‐1307, can be further promoted for cultivation for the study districts, while acc.217176b and acc.240478a deserve additional evaluation to expand varietal options for acidic soils in southern Ethiopia.

**TABLE 7:** Mean grain yield (t ha^−1^) performance of 10 barley genotypes tested in eight environments under acidic and lime‐treated soil conditions.

Genotype	Environment								Mean
	E1	E2	E3	E4	E5	E6	E7	E8	
G1	3.42^a^	3.76^a^	3.48^ab^	3.59^ab^	1.92^ab^	3.13^ab^	3.13^ab^	3.87^b^	3.29^b^
G2	2.42^d^	2.47^d^	2.65^e^	2.60^d^	1.06^d^	2.01^d^	1.80^d^	2.57^e^	2.20^e^
G3	2.96^b^	3.18^b^	3.33^bc^	3.39^bc^	1.65^a‐c^	2.88^bc^	2.36^c^	3.57^bc^	2.92^c^
G4	2.13^d^	2.34^d^	2.72^de^	2.81^d^	0.96^d^	2.02^d^	2.03^cd^	3.50^b‐d^	2.31^e^
G5	2.95^b^	3.18^b^	3.38^a‐c^	3.39^bc^	0.96^d^	2.17^d^	1.93^cd^	2.96^de^	2.62^d^
G6	2.13^d^	2.32^d^	2.64^e^	2.64^d^	1.07^d^	2.20^d^	2.35^c^	3.11^c‐e^	2.31^e^
G7	2.47^cd^	2.68^cd^	2.93^c–e^	3.07^cd^	1.55^bc^	2.81^bc^	2.94^b^	3.55^bc^	2.75^d^
G8	2.78^bc^	3.02^bc^	3.23^b–d^	3.43^bc^	1.30^cd^	2.52^cd^	2.34^c^	3.06^c‐e^	2.71^d^
G9	3.41^a^	3.69^a^	3.73^ab^	4.05^a^	1.99^a^	3.57^a^	3.52^a^	4.53^a^	3.56^a^
G10	2.95^b^	3.13^b^	3.90^a^	4.03^a^	1.83^ab^	2.97^bc^	3.18^ab^	3.97^ab^	3.24^b^
CV	4.2	4.5	5.7	5.0	8.9	7.6	6.3	5.7	5.84
*p*	< 0.0001	< 0.0001	< 0.0001	< 0.0001	< 0.0001	< 0.0001	< 0.0001	< 0.0001	< 0.0001

Abbreviations: E1 = Hulla, unlimed soil in 2021, E2 = Hulla, limed soil, 2021, E3 = Gumer, unlimed soil, 2021, E4 = Gumer, limed soil, 2021, E5 = Hulla, Unlimed soil, 2022, E6 = Hulla, Limed soil, 2022, E7 = Gumer, Unlimed soil, 2022, E8 = Gumer, Limed soil, 2022, G1 = 217176b, G10 = *Juma Tikur* (Local variety), G2 = 234911, G3 = 240478a, G4 = 240478b, G5 = 215453b, G6 = 215454a, G7 = 27895a, G8 = 208855b, G9 = HB‐1307 (released variety), means followed by a common superscript letter within a colomn are not significantly different from each other at P < 0.05.

### 3.4 AMMI Analysis of Variance for Grain Yield

The results on ANOVA of the AMMI model revealed that the environment, genotype, and GEI had highly significant effects (*p* < 0.001) on the grain yield performance of barley (Table 8). The environment's main effect accounted for 60.45% of the total variation, while the genotype and GEI accounted for 32.38% and 7.18% of the total variance, respectively. The GEI effect was further partitioned into various AMMI interaction principal component axes (AMMI‐IPCAs). Accordingly, the first four (AMMI‐PC1—AMMI‐PC4) were significant and accounted for 96.86% of the total GEI variance. The first two dependable and commonly used AMMI interaction principal component axes (AMMI‐IPCA1 and AMMI‐IPCA2) accounted for 61.65 and 18.95% of the total variance of GEI, respectively, and both together accounted for 80.59% of the total GEI variance. For the analysis of the biplots, both AMMI‐IPCA1 and AMMI‐IPCA2 are considered.

**TABLE 8:** Additive main effects and multiplicative interaction (AMMI) analysis of variance for grain yield of 10 barley genotypes evaluated in eight environments under acidic and lime‐treated soil conditions.

Sources of variation	df	SS	MS	Explained SS (%)			*F*
				Treatment	GEI	Cumulative	
Environment (E)	7	85.71	12.24	60.45		60.45	436.46***
Genotype (G)	9	45.91	5.10	32.38		92.82	181.82***
GEI	63	10.17	0.16	7.18		100.00	5.76***
AMMI‐PC1	15	6.27	0.42		61.65	61.65	14.73***
AMMI‐PC2	13	1.93	0.15		18.95	80.59	5.22***
AMMI‐PC3	11	1.01	0.09		9.92	90.51	3.23***
AMMI‐PC4	9	0.65	0.07		6.35	96.86	2.53**
AMMI‐PC5	7	0.27	0.04		2.63	99.49	1.35^NS^
AMMI‐PC6	5	0.03	0.01		0.32	99.80	0.23^NS^
AMMI‐PC7	3	0.02	0.01		0.20	100.00	0.24^NS^
Residuals	160	4.49	0.03				

Abbreviations: AMMI = Additive main effects and multiplicative interaction, df = degree of freedom, GEI = genotype by environment interaction, MS = mean square, NS = non‐significant, PC = principal component, SS = sum of squares.

** and *** significantly different at *p* < 0.01 and *p* < 0.001, respectively. ∗∗ and ∗∗∗ indicate significance at the 0.01 and 0.001 probability levels, respectively, and NS = not‐siginificant.

The AMMI model analysis further identified the top four‐grain yielding genotypes in each environment (Table 9). The highest grain‐yielding genotypes at E1 (Hulla, unlimed soil, 2021) were G1, G9, G3, and G5, while at E3 (Gumer, unlimed soil, 2021) the top performers included G10, G9, G1, and G5. The genotype G9 was the leading genotype in GY in the environment E4–E8, i.e., ranked first in these environments and second in the rest of the test environments (E1–E3). Likewise, G1 achieved the first place at E1 and E2, the second at E5 and E6, and the third at E3, E4, E7, and E8. Moreover, G5 consistently ranked as the fourth best performer at E1, E2, and E3, while G3 placed third at E1 and E2, and fourth at E6 and E8. Overall, the G9 was either the top or the second‐highest GY‐producing genotype across all environments, with G1 regularly presented among the top three. Similarly, G10, like G9 and G1, demonstrated strong grain yield performance, ranking first at E3, second at E4, and third at both E7 and E8 environments. Therefore, genotypes G9, G1, and G10 were identified as the most promising and selected genotypes based on their GY performance across the testing environments, followed by G3. In contrast, G5, G7, and G8 were recognized as fourth‐best performers in one or more environments (Table 9). Considering the first four top grain‐yielding genotypes, the environment, E8, was the highest in GY performance, followed by E4.

**TABLE 9:** The first four best barley genotypes selected for grain yield (t ha^−1^) by AMMI model per environment.

Environment	Code	Mean yield	IPCA1 score	Best genotypes			
				1	2	3	4
2021Hulla unlimed soil	E1	2.76	−0.798	G1	G9	G3	G5
2021 Hulla limed soil	E2	2.98	−0.781	G1	G9	G3	G5
2021 Gumer unlimed soil	E3	3.20	−0.572	G10	G9	G1	G5
2021 Gumer limed soil	E4	3.30	−0.407	G9	G10	G1	G8
2022 Hulla Unlimed soil	E5	1.43	0.251	G9	G1	G10	G7
2022 HullaLimed soil	E6	2.63	0.406	G9	G1	G10	G3
2022 Gumer Unlimed soil	E7	2.56	1.000	G9	G10	G1	G7
2022 Gumer Limed soil	E8	3.47	0.901	G9	G10	G1	G3
Grand mean		2.79					

Abbreviation: IPCA1 = the first interaction principal component axis.

### 3.5 AMMI‐1 Biplot for Grain Yield

**FIGURE 2:**
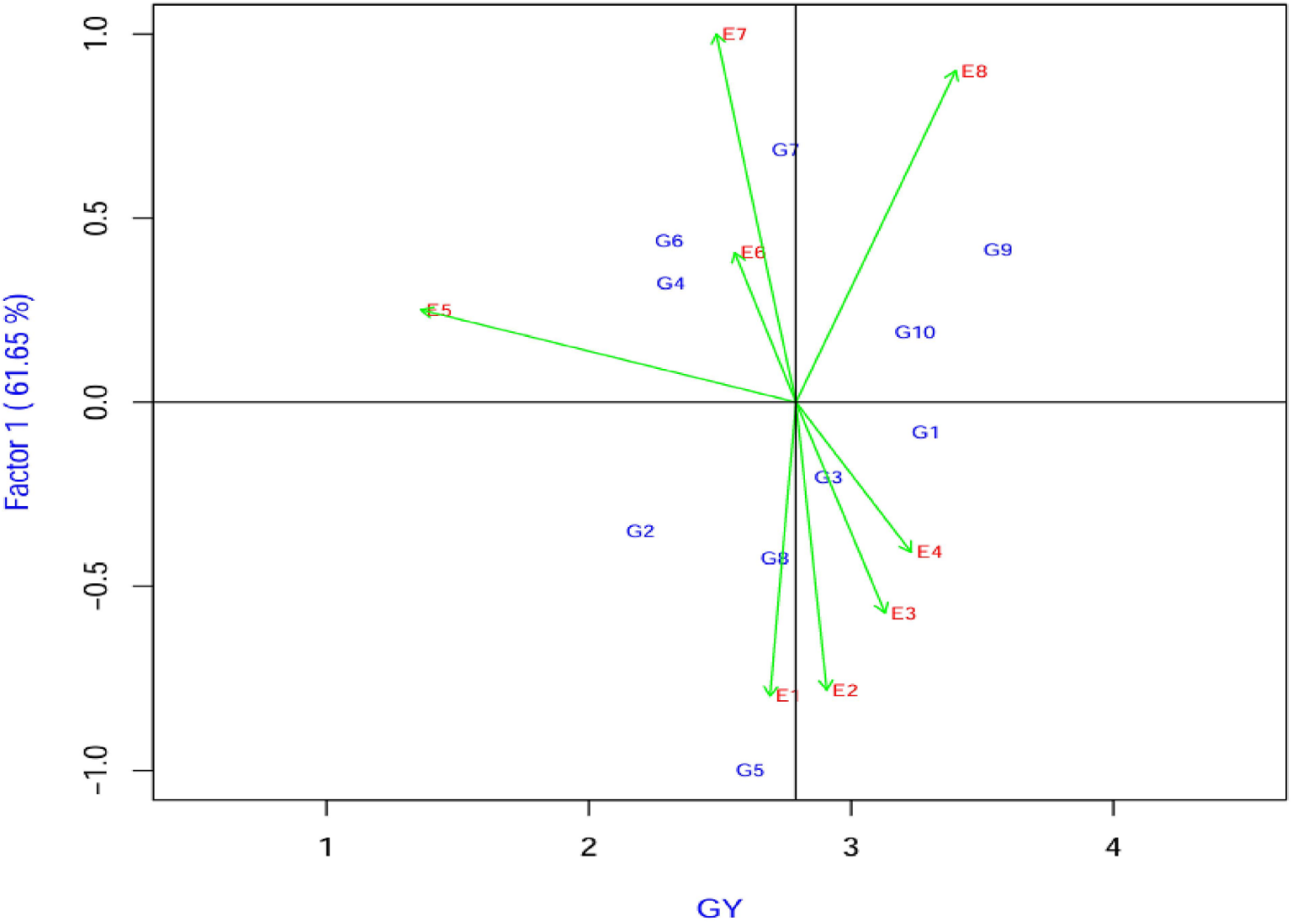
AMMI‐1 biplot of IPCA‐1 against the mean grain yield of 10 barley genotypes tested across eight environments under acidic soil conditions. G1 = 217176b; G2 = 234911; G3 = 240478a; G4 = 240478b; G5 = 215453b; G6 = 215454a; G7 = 27895a; G8 = 208855b; G9 = HB‐1307 (released variety); G10 = *Juma Tikur* (Local variety); E1 = Hulla, Unlimed soil in 2021; E2 = Hulla, Limed soil, 2021; E3 = Gumer, Unlimed soil, 2021; E4 = Gumer, limed soil, 2021; E5 = Hulla, Unlimed soil, 2022; E6 = Hulla, Limed soil, 2022; E7 = Gumer, Unlimed soil, 2022; E8 = Gumer, Limed soil, 2022.

In the AMMI‐1 biplot analysis, the primary effects of genotypes and environments, along with the IPCA‐1 scores, are depicted in a comparative plot (Figure 2). The IPCA‐1 from the AMMI‐1 accounted for 61.65% of the total variance in the interaction effects. The results revealed a wide range of environments, with E3, E4, and E6 plotted close to the origin of the biplot, while E1, E2, and E5 were located at relatively intermediate distances. In contrast, E7 and E8 had longer vector lengths than the others and exhibited high positive IPCA‐1 scores. The AMMI‐1 biplot indicated a more scattered pattern of the environments than the genotypes. Most genotypes are located close to the origin of the biplot, except for G5, followed by G7, which are far from the origin. Genotypes G1, G3, and G10 are positioned nearer to the zero of the IPCA‐1 score. The remaining genotypes are located at relatively intermediate distances from the zero IPCA‐1 score. According to the AMMI‐1 biplot for IPCA‐1, genotypes G4, G6, G7, G9, and G10 demonstrate positive interaction effects at E5, E6, E7, and E8, but exhibit negative interaction in the opposite environments with a negative IPCA‐1 score. Conversely, the other genotypes and environments show the opposite pattern.

### 3.6 AMMI‐2 Biplot for Grain Yield

**FIGURE 3:**
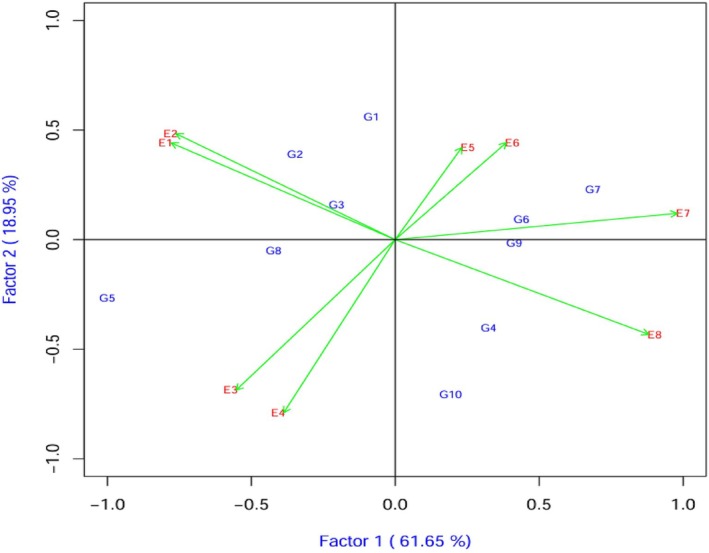
AMMI‐2 biplot of IPCA‐1 against IPCA‐2 for mean grain yield of 10 barley genotypes tested across eight environments under acidic soil conditions. G1 = 217176b; G2 = 234,911; G3 = 240478a; G4 = 240478b; G5 = 215453b; G6 = 215454a; G7 = 27895a; G8 = 208855b; G9 = HB‐1307 (released variety); G10 = *Juma Tikur* (Local variety); E1 = Hulla, unlimed soil in 2021; E2 = Hulla, limed soil, 2021; E3 = Gumer, unlimed soil, 2021; E4 = Gumer, limed soil, 2021; E5 = Hulla, unlimed soil, 2022; E6 = Hulla, limed soil, 2022; E7 = Gumer, unlimed soil, 2022; E8 = Gumer, limed soil, 2022.

The AMMI‐2 biplot analysis indicated that the first interaction principal component scores for both the genotypes and environments were plotted against the second interaction principal component scores for the genotypes and environments (Figure 3). In this biplot, IPCA1 and IPCA2 accounted for 61.65% and 18.95% of the total interaction variance, respectively, with highly significant effects. Thus, the first two IPCAs together explained 80.6% of the total interaction variance. Both the environments and genotypes were scattered in the biplot at varying distances from the origin. Accordingly, E5 and E6 were closer to the origin of the biplot, indicating moderate discrimination with good representativeness, making them efficient sites for future testing. Comparatively, E1, E2, E3, and E4 had intermediate distances from the origin, with E1 and E2 reflecting lower variation or appearing with similar environmental effects. Among the environments, E7 and E8 exhibited the longest vectors, thus contributing most to the GEI compared to the others. Genotypes G3, G9, G6, G8, and G4 are plotted close to the origin of the biplot, indicating broad adaptation and stability. Genotypes G1, G4, G7, and G10 were intermediate, and G5 showed the greatest negative IPCA1 score, reflecting narrow adaptation under low‐yielding conditions, and this was followed by G7. The first two IPCAs were positive for genotypes G6 and G7 and environments E5 and E6, whereas the genotypes G5 and G8 with E3 and E4 had negative scores for the first two IPCAs. In contrast, genotypes G1, G2, and G3, along with E1 and E2, tested positive for the first two IPCAs and negative for the second two IPCAs.

## 4 Discussion

### 4.1 Analysis of Variance

The genotypic, environmental, and the interaction effects influenced barley agronomic and phenological traits and grain yield stability. The genotypes performed variably across the test environments that varied in climate, altitude, soil properties, and abiotic factors. Maniruzzaman et al. (2019), Nehe et al. (2019) and Kebede et al. (2023) also report the influence of environments on crop performance. Some of the soil environments were treated with lime (CaCO_3_), which further enhanced environmental effects in addition to the natural variability through modifying the soil properties and nutrient availability, as reported in research findings by Caires et al. (2006), Paradelo et al. (2015). The genetic variability among the genotypes also led to significant differences in the measured traits. Our result aligns with Abtew et al. (2015), who found wider variation among Ethiopian barley genotypes in their agronomic performances. The highly significant effect of the GEI highlighted the need for careful evaluation and selection of genotypes for particular environments in the region. Our findings are consistent with Maniruzzaman et al. (2019), who also observed substantial variations in relation to genotype, environment, and their interaction effects on the crop performances.

### 4.2 Effect of Environments on Barley Performance

The test environments in this study were categorized as either limed or unlimed soil conditions. Lime application, in conjunction with the inherent soil and other environmental variation, significantly (*p* < 0.05) influenced the performance of individual genotypes. Previous studies have also documented the impact of environmental variation on crop performance (Gauch and Zobel 1996; Abtew et al. 2015; Verma et al. 2016; Kebede et al. 2023). Environments E4 and E8 showed favorable performance across most measured traits, supporting improved growth, grain yield, and yield components. Their superior performance was likely associated with lower initial soil acidity and the application of lime at 70% and 100% of the recommended rates, respectively. In general, all limed environments, except the E6, produced grain yields above the grand mean and outperformed their corresponding unlimed environments. Although E6 recorded relatively low yields, likely due to limited early‐season rainfall, lime application still increased genotype performance by 183.91% compared with the E5, which had similar geographic, climatic, and soil conditions but received no lime. These findings indicate that liming enhanced the discriminating ability of the test environments by improving soil conditions that favored crop growth and yield. Soil acidity associated with aluminum (Al^3+^) toxicity is a major constraint to barley production, particularly in high‐rainfall areas (Abdenna et al. 2007). Under acidic soil conditions, Al^3+^ toxicity inhibits root growth by restricting cell division and elongation, thereby reducing water and nutrient uptake and limiting crop productivity (Wang et al. 2006). Liming is therefore widely recommended to increase soil pH, alleviate aluminum toxicity, and improve nutrient availability in acidic soils (Desalegn et al. 2017; Demissie et al. 2017). The beneficial effects of lime are primarily attributed to increased soil pH and the associated improvements in soil conditions that promote crop growth (Haynes and Ludecke 1981).

Several studies have documented the positive effects of liming on barley production in the acidic soils of Ethiopia. By raising soil pH, reducing aluminum toxicity, and increasing the availability of essential nutrients, liming creates more favorable conditions for barley growth (Chimdi et al. 2012; Alemu et al. 2017; Desalegn et al. 2017). The poor performance of crops in the unlimed soils observed in the present study is consistent with the findings of Elisa et al. (2016). Overall, both environmental factors and lime application influenced the variation in barley performance across the test environments, with liming exerting a significant positive effect on productivity in the acidic soils of the study districts.

### 4.3 Mean Performance of Genotypes

Barley genotypes exhibited significant variation across all the measured agronomic and phenology traits. Their performance varied among the evaluated parameters, and no single genotype was superior for all the assessed traits. For example, genotype G1 performed best for most growth and yield‐related traits, whereas thousand‐seed weight (TSW) was statistically highest in G2 and G3, followed by G5 among the accessions used. The check cultivar G9 (HB‐1307) and the local variety G10 (*Juma Tikur*) exhibited the longest days to flowering and maturity compared with the accessions. Overall, G1 (acc. 217176b), G9, and G10 demonstrated superior performance across multiple agronomic and phenology traits. While genotype G1 and G10 are promising candidates for further evaluation, the results support the wider promotion and adoption of the released cultivar HB‐1307 in the study districts to sustainably enhance barley production and household food security.

Genotype rankings varied significantly across the test environments for their grain yields, indicating differential responses to environmental conditions. An exception was observed in environment E5, where all genotypes performed poorly and showed no interaction, resulting in no changes in the rank. These findings highlight the importance of identifying specific environments that are best suited for a particular group of genotypes in the study districts. The results of our findings were in line with those of Mulatu and Grando (2006), and Abtew et al. (2015), who indicated that Ethiopian barley genotypes exhibited wide variation in their agronomic and phenological responses to different environments.

GEI had a highly significant (*p* < 0.001) effect on most measured traits, including grain yield. Genotype G10, a widely cultivated local variety in acid‐prone areas of Gumer district, and acc. 217176b (G1), a high‐performing accession, showed strong competitiveness with the check/released cultivar HB‐1307 (G9). Their stable performance under both limed and unlimed soil conditions highlights their potential for breeding and seed expansion. Particularly, despite its apparent tolerance to soil acidity, acc. 217176b has not yet been adopted by farmers in the region, and the seeds were conserved at Ethiopian Biodiversity Institute (EBI). The superior performance of these genotypes may be attributed to their ability to tolerate acidic soil conditions and efficiently utilize nutrients from both native and applied sources. Similar genotype‐specific responses to soil acidity have been reported by Zhao et al. (2003) and Sisay and Balemi (2014). Consistent with previous studies by Obata and Fernie (2012) and Fekadu et al. (2022), our findings demonstrate that genetic variation plays a critical role in barley adaptation to acidic soils and influences yield performance through interactions with environmental conditions. Significant variation in phenology traits was also observed among genotypes, supporting the importance of genetically controlled phenology diversity in selection and breeding programs, as reported by Jankowicz‐Cieslak et al. (2017). Although the genotypes differed in their phenological traits due to genetic variation, all test genotypes flowered and matured earlier under the limed soils than under the unlimed acidic soil conditions. Therefore, liming reduced the duration of the phenological stages. Earlier maturity is particularly important in the highlands of both the study districts, as it allows barley to be harvested before the onset of *Belg* rainy season (short rainy season), enabling double cropping and improving land productivity.

### 4.4 Barley Grain Yield Evaluation Based on AMMI

The AMMI analysis of variance revealed highly significant (*p* < 0.001) effects of environment, genotype, and GEI on grain yield. Among these sources of variation, the environment accounted for the largest proportion (60.45%), followed by genotype (32.38%) and GEI (7.18%). The considerable impact of the environment on the overall grain yield variation suggests significant variability among the test environments. This variation can be attributed to the inherent differences among the environments as well as the effects of lime treatment on some of the selected environments. Thus, liming helped in better distinguishing the environments for identifying genotypes that are tolerant to soil acidity stress. Various studies also indicated that a large proportion of the variations attributed to environments (Gauch and Zobel 1997; Rodriguez et al. 2008; Solonechnyi et al. 2015; Kebede et al. 2023). Following the environment, the higher share of the genotypes' effect on the total variation indicated wider genetic differences among the test genotypes in grain yield performance across the test environments. In other words, unlike many researchers report (Rodriguez et al. 2008; Kebede et al. 2023), the GEI contribution to the total variation in grain yield performances was the lowest. Yan and Kang (2003) indicated that large GEI, relative to genotype effect suggests the possible existence of different mega‐environments with different top‐yielding genotypes. In this study, a consistent group of high‐yielding genotypes showed minimal variation in grain yield within themselves, leading to a low contribution of GEI to total variation. However, regardless of the third position in contributing to the total variation, the GEI effect was highly significant (*p* < 0.001).

The GEI effect was partitioned into AMMI interaction principal component axes (IPCAs), and the results showed that the first two IPCAs accounted for most of the grain yield variation among barley genotypes. This finding agrees with previous studies (Zobel et al. 1988; Legesse et al. 2015; Tena et al. 2019), which reported that the first two IPCAs capture a substantial proportion of GEI variation. Similarly, Gauch et al. (2008) and Fekadu et al. (2022) indicated that the first two IPCAs are sufficient for explaining GEI patterns, assessing genotype stability, and constructing reliable two‐dimensional AMMI biplots. In contrast, subsequent IPCAs mainly represent non‐predictive random variation and contribute little to predictive accuracy (Kaya et al. 2002). Therefore, only the first two IPCAs were considered for the GEI interpretation in the current study.

Based on the AMMI model analysis, the test environments are categorized into favorable and unfavorable environments for barley production. A favorable environment has a mean yield above the grand mean of all environments, while an unfavorable environment has a mean yield below the grand mean (Matlala et al. 2019; Kindie et al. 2021). Thus, environments E2, E3, E4, and E8 were considered as favorable environments while E1, E5, E6, and E7 were considered as unfavorable environments to cultivate the tested barley genotypes for grain yield production. Among the environments, E8 was the highest with 24.4% above the grand mean while E5 was the most unfavorable environment with 51.3% less grain yield production compared to the grand mean. The greater reductions in the grain yield observed at E5 are likely attributable to higher initial soil acidity, the absence of lime treatment, and suboptimal rainfall during the early growth stages of the genotypes in this environment. Based on grain yield analysis using the AMMI model, the genotypes G1, G3, G9, and G10 demonstrated suitability for cultivation in both the favorable and unfavorable environments. In contrast, genotype G7 exhibited superior performance specifically in the unfavorable environments, whereas G8 was more productive in the favorable environments.

### 4.5 Grain Yield Evaluation Based on AMMI‐1

In the AMMI‐1 biplot analysis, grain yield was plotted against the first IPCA scores of genotypes and environments. This approach helps visualize the GEI (Kaya et al. 2002). The vertical line (ordinate) shows the grand mean yield. The horizontal line (abscissa) at IPCA‐1 scores indicates the interaction pattern. Genotypes and environments to the right of the ordinate are high yielding. Those to the left are low yielding (Erdemcı 2018; Ngeve and Bouwkamp 1993). In this study, genotypes G9, G10, G1, and G3, as well as environments E8, E4, E3, and E2, were to the right of the ordinate. This indicates superior performance. Conversely, all remaining genotypes and environments fell below the grand mean yield. Genotypes and environments along the same vertical (ordinate) line have similar yield levels. Those along the same horizontal (abscissa) line show similar interaction patterns (Fekadu et al. 2022; Kilic 2014). Thus, G10 and G1 yielded similarly, while G6 and G9 shared a common interaction pattern.

Genotypes and environments with similar first IPCA score signs exhibit positive interactions, indicating favorable conditions for those genotypes within those environments (Yohane et al. 2021). To apply this, dividing the target environments into meaningful mega‐environments and deploying different cultivars for each mega‐environment is the recommended approach. This allows positive GEI to be exploited and negative GEI to be avoided (Yan and Tinker 2005). Therefore, the high‐yielding genotypes, such as G9 and G10, showed positive IPCA‐1 scores, suggesting a beneficial interaction with the environment E8 with greater yield production under fully limed soil environments. This implies that liming is particularly suitable for these genotypes, while the E1 environment, which is positioned on the opposite side, is unfavorable for them. Conversely, other high grain‐yielding genotypes like G1 and G3 demonstrated negative IPCA‐1 scores when associated with the E2, E3, and E4 environments. This indicated a positive interaction with these environments, making them more favorable, while E5 and E6, located on the opposite side, are less suitable for G1 and G3. Among the genotypes examined, G9 and G2 were situated relatively far from the grand mean. In contrast, the other genotypes were closer to the grand mean, suggesting they contributed less to the overall variation (Abraha et al. 2019). Moreover, the environments displayed considerable scatter on the biplot, indicating that the environmental contribution to total variation was greater than that of the genotypes' effect (Kebede et al. 2023).

Selection of genotypes should account for both stability and grain performance (Movahedi et al. 2020). The magnitude of a genotype's IPCA‐1 score on the biplot shows its contribution to GEI variance (Kilic 2014). Genotypes with IPCA‐1 scores close to zero on the abscissa contribute less to the GEI variation. This means they demonstrate stable performance. In contrast, genotypes with high IPCA‐1 scores, located far from zero on the *x*‐axis, contribute more to the GEI variance and are unstable across environments. Ebdon and Gauch (2002) indicated that genotypes with IPCA‐1 scores near zero have wide adaptation, while those with high scores have specific adaptation. In the current study, genotypes G3, G1, and G10 had grain yields above the grand mean and low IPCA‐1 scores. These genotypes contributed less to the GEI variance and performed consistently across environments. Genotypes G4, G6, G8, G2, and G9 had intermediate IPCA‐1 scores, suggesting relatively stable performance across environments. Among these, G9 had the highest yield and was far from the *Y*‐axis, close to E8. This indicates that G9 made a significant contribution to the GEI variation and is optimally adapted to environment E8, where the soil was adequately treated with lime. In contrast, genotypes G5 and G7 had high IPCA‐1 scores. This indicates they had substantial impacts on the overall GEI variance. They also showed greater variability in grain yield performance across the different environments.

The contribution of environments to the total GEI variance varied significantly. The magnitude of these contributions was assessed using the vector lengths of the environments (Fekadu et al. 2022). Environments E7, E8, and E5 exhibited longer vectors. This demonstrated their high discriminating power and significant contributions to the total GEI variance. Conversely, environments E1 and E2 showed intermediate discriminating abilities and moderate contributions to GEI. Environments with longer vectors (E7, E8, and E5), which demonstrate better discrimination capabilities, were considered more suitable for testing genotypes. This is particularly true when resources are limited and conducting multi‐environment yield trials at only a few locations is necessary (Daba et al. 2015). In contrast, environments E6, E4, and E3 had relatively shorter vectors. This indicated low discriminating abilities concerning genotype performance for grain yield and contributed minimally to the total variation of the GEI.

### 4.6 Grain Yield Evaluation Based on AMMI‐2

In the AMMI‐2 biplot analysis, the first and second Interaction Principal Component Axis (IPCA) scores were plotted to assess the GEI magnitude. Genotypes closer to the biplot origin are more stable and less influenced by environmental interactions, while those farther away are less stable (Purchase 1997). Gauch and Zobel (1996) and Ferney et al. (2006) stated that IPCA2 scores near zero indicate greater stability across environments. In this study, genotypes G3, G9, G8, and G6 were closest to the origin, reflecting higher stability. Genotypes G2, G4, G7, G10, and G1 showed intermediate stability, while G5, which was farthest from the origin, was the least stable. Genotypes and environments in close proximity on the biplot share similar interaction patterns, while those farther apart differ. Hussain et al. (2005) observed that genotypes closely associated with specific environments demonstrate targeted stability. For instance, G6 and G7 performed well in environments E6, E7, and E5, but not in E3 and E4, which are positioned on the opposite side of the biplot. Similarly, G9, G4, and G10 performed well in E8 but poorly in E1 and E2 due to differing interactions.

The stability performances of the genotypes varied between the AMMI‐1 and AMMI‐2 biplot analyses. For instance, G3, G1, and G10 yielded above the grand mean and showed better stability performance in AMMI‐1. However, in AMMI‐2, G1 and G10 were far from the origin indicating less stability. Genotype G3 had a shorter distance in AMMI‐1 and maintained a short distance in AMMI‐2. Other genotypes, such as G9, G6, G8, and G4, also had shorter distances in AMMI‐2. This indicated that they had better stability than G1 and G10. Tolessa et al. (2013) and Oliveira et al. (2014) emphasized that AMMI‐2 is more stringent and accurate than AMMI‐1. This is because AMMI‐2 accounts for a higher total variation of the GEI. Environments E7 and E8 displayed greater vector distances from the biplot origin. This highlights their effectiveness in distinguishing genotype performance for grain yield compared to the other test environments. In contrast, E5 and E6 were closer to the biplot origin, indicating lower discriminating power regarding genotypes for grain yield. Environments E1 to E4 had moderate distances from the biplot origin. These showed average discriminating ability concerning barley genotype performance for grain yield. Genotypes and environments exhibit similar interaction patterns when both have the same sign for IPCA‐1 and IPCA‐2 scores. When genotypes have different signs for the first two IPCA scores compared to the environments, they display differing interaction patterns, often resulting in a negative interaction. For example, G3, G2, and G1 interact positively with environments E1 and E2. Whereas, E8, located on the opposite side of the biplot, exhibited negative interactions with those genotypes. Similarly, G6 and G7 interact positively with environments E5, E6, and E7, but negatively with E3 and E4. From an agronomic perspective, G9, G10, G1, and G3 were the highest‐yielding genotypes, while G3, G9, G6, G8, and G4 showed the greatest yield stability across environments. Both genotypes, G3 and G9, combined high yield with broad adaptation and stability, whereas G1 and G10 achieved high yields but were more specifically adapted. Since grain yield is the main production goal and acid‐soil‐tolerant seed sources are limited, G9 (HB‐1307) can be widely promoted for farmer adoption, and G1 and G3 are recommended for further evaluation and seed registration.

The cosine of the angle between the vectors of two environments quantifies the genetic correlation between them (Kroonenberg 1995; Yan 2001). This metric facilitates the visualization of genotype ranking similarities across environments. The spatial distance between environment vectors is therefore essential for identifying significant variability within the test group. For example, pairs of environments i.e., E1 and E2, E5 and E6, and E3 and E4 are plotted in close proximity, forming acute angles (Figure 3). This spatial closeness indicates that these pairs of environments provide similar information, reflecting a positive correlation in genotype responses. Daba et al. (2015) reported that a close association among test locations enables the extraction of comparable genotype information from fewer sites, which may reduce testing costs.

## 5 Conclusion

Environment, genotype, and their interaction significantly affected all agronomic and phenological traits of barley grown in acidic soils. The environment explained the largest proportion of variation, followed by genotype and GEI. Lime application improved growing conditions, increased genotype differentiation among environments, and enhanced barley growth and grain yield. Environment E8, with low initial soil acidity and the full lime rate, produced the highest grain yield, 24% above the grand mean. In contrast, E5, characterized by high soil acidity and no lime application, recorded the lowest yield, 49% below the grand mean. Genotypes responded differently to environmental conditions and GEI, although high‐yielding genotypes generally showed greater stability. The AMMI analysis identified G9, G10, G1, and G3 as high‐yielding genotypes, while G3, G9, G6, G8, and G4 were the most stable. Among these, G3 and G9 combined high yield with broad adaptation and stability, whereas G1 and G10 showed moderate adaptation. The AMMI model indicated that G1, G3, G9, and G10 performed well across favorable and unfavorable environmental conditions. Both G1 and G3 were collected accessions, G10 was a local control, and G9 was a released variety adapted to the Ethiopian mid‐ and highlands, although it remains underutilized by local farmers. The current study identified G9 as a better‐performing released variety, G1 and G3 as promising accessions, and G10 as a potential local variety for acidic soils in southern Ethiopia. Therefore, wider adoption of the improved variety HB‐1307 (G9), further evaluation and possible release of accession 217176b (G1), 240478a (G3), and the local variety (Juma Tikur), together with lime application based on the exchangeable acidity levels, could improve barley productivity and contribute to food security in the region.

## Funding

This study was funded by the thematic research grant of Hawassa University, Ethiopia.

## Conflicts of Interest

The authors declare no conflicts of interest.

## Supporting information


**Data S1:** Data used for AMMI analysis.

## Data Availability

Data available in article Supporting Information.

## References

[pei370192-bib-0001] Abdenna, D. , C. Negassa , and G. Tilahun . 2007. “Inventory of Soil Acidity Status in Crop Lands of Central and Western Ethiopia.” Utilisation of Diversity in Land Use Systems: Sustainable and Organic Approaches to Meet Human Needs, Tropentag.

[pei370192-bib-0002] Abraha, M. T. , H. Shimelis , T. Solomon , and A. Hailu . 2019. “Genotype‐by‐Environment Interaction and Selection of Elite Wheat Genotypes Under Variable Rainfall Conditions in Northern Ethiopia.” Journal of Crop Improvement 33, no. 6: 797–813.

[pei370192-bib-0003] Abtew, W. G. , B. Lakew , B. I. Haussmann , and K. J. Schmid . 2015. “Ethiopian Barley Landraces Show Higher Yield Stability and Comparable Yield to Improved Varieties in Multi‐Environment Field Trials.” Journal of Plant Breeding and Crop Science 7, no. 8: 275–291.

[pei370192-bib-0004] Agegnehu, G. , C. Yirga , and T. Erkossa . 2019. Soil Acidity Manageme*Nt. Vol. 21. Ethiopian Institute of Agricultural Research (EIAR).

[pei370192-bib-0005] Ahmed, M. , S. Ahmad , G. Abbas , S. Hussain , and G. Hoogenboom . 2024. Cropping Systems Modeling Under Changing Climate. Springer.

[pei370192-bib-0006] Alemu, G. , T. Desalegn , T. Debele , A. Adela , G. Taye , and C. Yirga . 2017. “Effect of Lime and Phosphorus Fertilizer on Acid Soil Properties and Barley Grain Yield at Bedi in Western Ethiopia.” African Journal of Agricultural Research 12, no. 40: 3005–3012.

[pei370192-bib-0007] Andersson, S. , and S. I. Nilsson . 2001. “Influence of pH and Temperature on Microbial Activity, Substrate Availability of Soil‐Solution Bacteria and Leaching of Dissolved Organic Carbon in a Mor Humus.” Soil Biology and Biochemistry 33, no. 9: 1181–1191.

[pei370192-bib-0008] Annicchiarico, P. 2002. Genotype × Environment Interactions: Challenges and Opportunities for Plant Breeding and Cultivar Recommendations. Food and Agriculture Organization of the United Nations.

[pei370192-bib-0009] Aweke, M. G. 2017. Climate‐Smart Agriculture in Ethiopia: CSA Country Profiles for Africa Series. International Center for Tropical Agriculture.

[pei370192-bib-0010] Bavandpori, F. , J. Ahmadi , and S. M. Hossaini . 2015. “Yield Stability Analysis of Bread Wheat Lines Using AMMI Model.” Agricultural Communications 3, no. 1: 8–15.

[pei370192-bib-0011] Bilgin, O. , A. Balkan , Z. K. Korkut , and İ. Başer . 2018. “Multi‐Environmental Evaluation of Triticale, Wheat and Barley Genotypes by GGE Biplot Analysis.” Journal of Life Sciences 12: 13–23.

[pei370192-bib-0012] Bizuneh, W. F. , and D. A. Abebe . 2019. “Malt Barley ( *Hordeum distichon* L.) Varieties Performance Evaluation in North Shewa, Ethiopia.” African Journal of Agricultural Research 14, no. 8: 503–508.

[pei370192-bib-0013] Caires, E. F. , G. Barth , and F. J. Garbuio . 2006. “Lime Application in the Establishment of a No‐Till System for Grain Crop Production in Southern Brazil.” Soil and Tillage Research 89, no. 1: 3–12.

[pei370192-bib-0014] Central Statistics Agency (CSA) . 2017. Agricultural Sample Survey: Report on Area and Production of Major Crops (Private Peasant Holdings, Meher Season), 2016/2017 (2009 E.C.), Volume I, Statistical Bulletin 584. Central Statistical Agency (CSA).

[pei370192-bib-0015] Chimdi, A. , H. Gebrekidan , K. Kibret , and A. Tadesse . 2012. “Response of Barley to Liming of Acid Soils Collected From Different Land Use Systems of Western Oromia, Ethiopia.” Journal of Biodiversity and Environmental Sciences 2, no. 7: 1–13.

[pei370192-bib-0016] Crossa, J. , H. G. Gauch Jr. , and R. W. Zobel . 1990. “Additive Main Effects and Multiplicative Interaction Analysis of Two International Maize Cultivar Trials.” Crop Science 30, no. 3: 493–500.

[pei370192-bib-0017] Daba, C. , A. Ayana , H. Zeleke , and A. Wakjira . 2015. “Genotype × Environment Interactions for Seed Yield in Sesame in Western Ethiopia.” East African Journal of Sciences 9, no. 2: 85–96.

[pei370192-bib-0018] Demissie, W. , S. Kidanu , and V. R. Cherukuri . 2017. “Effect of Integrated Use of Lime, Blended Fertilizer and Compost on Productivity, Nutrient Removal and Economics of Barley ( *Hordeum vulgare* L.) on Acid Soils of High Lands in West Showa Zone of Ethiopia.” International Journal of Life Sciences 5, no. 3: 311–322.

[pei370192-bib-0019] Desalegn, T. , G. Alemu , A. Adella , and T. Debele . 2017. “Effect of Lime and Phosphorus Fertilizer on Acid Soils and Barley ( *Hordeum vulgare* L.) Performance in the Central Highlands of Ethiopia.” Experimental Agriculture 53, no. 3: 432–444.

[pei370192-bib-0020] Diriba, G. 2020. “Ethiopian Economics Association (EEA).”

[pei370192-bib-0021] Doehlert, D. C. , M. S. McMullen , and J. J. Hammond . 2001. “Genotypic and Environmental Effects on Grain Yield and Quality of Oat Grown in North Dakota.” Crop Science 41, no. 4: 1066–1072.

[pei370192-bib-0022] Ebdon, J. S. , and H. G. Gauch Jr. 2002. “Additive Main Effect and Multiplicative Interaction Analysis of National Turfgrass Performance Trials: I. Interpretation of Genotype× Environment Interaction.” Crop Science 42, no. 2: 489–496.

[pei370192-bib-0023] Elisa, A. A. , S. Ninomiya , J. Shamshuddin , and I. Roslan . 2016. “Alleviating Aluminum Toxicity in an Acid Sulfate Soil From Peninsular Malaysia by Calcium Silicate Application.” Solid Earth 7, no. 2: 367–374.

[pei370192-bib-0024] Erdemcı, İ. 2018. “Investigation of Genotype× Environment Interaction in Chickpea Genotypes Using AMMI and GGE Biplot Analysis.” Turkish Journal of Field Crops 23, no. 1: 20–26.

[pei370192-bib-0025] Etana, D. , and D. Merga . 2021. “Additive Main Effect and Multiplicative Interaction Model (AMMI) in Plant Breeding Stability Analysis: Review.” Journal of Agricultural Research Pesticides and Biofertilizers 2, no. 3: 1–7.

[pei370192-bib-0026] Evans, O. , L. Dickson , M. Thomas , et al. 2013. “Enhancing Maize Grain Yield in Acid Soils of Western Kenya Using Aluminum Tolerant Germplasm.” Journal of Agricultural Science and Technology. A 3, no. 1A: 33.

[pei370192-bib-0027] Eyasu, E. 2016. Soils of the Ethiopian Highlands: Geomorphology and Properties, 385. CASCAPE Project, ALTERA, Wageningen University and Research Centre (Wageningen UR). https://bit.Ly/3s4T3jO.

[pei370192-bib-0029] Fekadu, E. , K. Kibret , and A. Melese . 2019. “Integrated Acid Soil Management for Growth, Nodulation, and Nutrient Uptake of Faba Bean ( *Vicia faba* L.) in Lay Gayint District, Northwestern Highlands of Ethiopia.” International Journal of Agronomy 2019, no. 1: 7498518.

[pei370192-bib-0030] Fekadu, W. , F. Mekbib , B. Lakew , and B. I. Haussmann . 2022. “Assessment of Genetic Variability and Acid Soil Tolerance in Ethiopian Barley Landraces.” Ethiopian Journal of Agricultural Sciences 32, no. 4: 1–29.

[pei370192-bib-0032] Ferney, G. B. , M. Alexei , and A. Aigul . 2006. “Evaluation of Grain Yield Stability, in Spring Wheat From Kazakhstan and Siberia.” Journal of Central European Agriculture 7: 649–660.

[pei370192-bib-0033] Foy, C. D. 1988. “Plant Adaptation to Acid, Aluminum‐Toxic Soils.” Communications in Soil Science and Plant Analysis 19, no. 7–12: 959–987.

[pei370192-bib-0034] Gauch, H. G., Jr. 2006. “Statistical Analysis of Yield Trials by AMMI and GGE.” Crop Science 46, no. 4: 1488–1500.

[pei370192-bib-0035] Gauch, H. G. 2007. “MATMODEL Version 3.0: Open Source Software for AMMI and Related Analyses.” Crop and Soil Sciences, Cornell University, Ithaca, NY.

[pei370192-bib-0036] Gauch, H. G., Jr. , H. P. Piepho , and P. Annicchiarico . 2008. “Statistical Analysis of Yield Trials by AMMI and GGE: Further Considerations.” Crop Science 48, no. 3: 866–889.

[pei370192-bib-0037] Gauch, H. G. , and R. W. Zobel . 1996. “AMMI Analysis of Yield Trials.” In Genotype by Environment Interaction, edited by M. S. Kang and H. G. Gauch , 85–122. CRC Press.

[pei370192-bib-0100] Gauch, H. G., Jr. , and R. W. Zobel . 1997. “Identifying Mega‐environments and Targeting Genotypes.” Crop Science 37, no. 2: 311–326.

[pei370192-bib-0038] Gauch, H. J. 1992. “Statistical Analysis of Regional Yield Trials: AMMI Analysis of Factorial Designs.” pp. xi+−278.

[pei370192-bib-0098] Gauch, H. J. 1992. Statistical Analysis of Regional Yield Trials: AMMI Analysis of Factorial Designs, xi+–278. Elsevier Science Publishers.

[pei370192-bib-0039] Giraldo, P. , E. Benavente , F. Manzano‐Agugliaro , and E. Gimenez . 2019. “Worldwide Research Trends on Wheat and Barley: A Bibliometric Comparative Analysis.” Agronomy 9, no. 7: 352.

[pei370192-bib-0040] Gollob, H. F. 1968. “A Statistical Model Which Combines Features of Factor Analytic and Analysis of Variance Techniques.” Psychometrika 33, no. 1: 73–115. 5239571 10.1007/BF02289676

[pei370192-bib-0041] Gomez, K. A. , and A. A. Gomez . 1984. Statistical Procedures for Agricultural Research. 2nd ed. International Rice Research Institute, John Wiley and Sons Inc.

[pei370192-bib-0042] Grover, S. P. , C. R. Butterly , X. Wang , and C. Tang . 2017. “The Short‐Term Effects of Liming on Organic Carbon Mineralisation in Two Acidic Soils as Affected by Different Rates and Application Depths of Lime.” Biology and Fertility of Soils 53, no. 4: 431–443.

[pei370192-bib-0043] Hartley, H. O. 1950. “The Maximum F‐Ratio as a Short‐Cut Test for Heterogeneity of Variance.” Biometrika 37, no. 3/4: 308–312. 14801057

[pei370192-bib-0099] Haynes, R. J. , and T. E. Ludecke . 1981. “Effect of Lime and Phosphorus Applications on Concentrations of Available Nutrients and on P, Al and Mn Uptake by Two Pasture Legumes in an Acid Soil.” Plant and Soil 62, no. 1: 117–128.

[pei370192-bib-0044] Hussain, A. , S. Khan , and M. Bashir . 2005. “Influence of Environment on Yield Related Traits of Exotic Oats Cultivars.” Sarhad Journal of Agriculture 21, no. 2: 209–213.

[pei370192-bib-0045] Jankowicz‐Cieslak, J. , C. Mba , and B. J. Till . 2017. “Mutagenesis for Crop Breeding and Functional Genomics.” In Biotechnologies for Plant Mutation Breeding: Protocols, 3–18. Springer.

[pei370192-bib-0046] Jeberson, M. S. , L. Kant , N. Kishore , V. Rana , D. P. Walia , and D. Singh . 2017. “AMMI and GGE Biplot Analysis of Yield Stability and Adaptability of Elite Genotypes of Bread Wheat ( *Triticum aestivum* L.) for Northern Hill Zone of India.” International Journal of Bio‐Resource and Stress Management 8, no. 5: 635–641.

[pei370192-bib-0047] Kaya, Y. , C. Palta , and S. Taner . 2002. “Additive Main Effects and Multiplicative Interactions Analysis of Yield Performances in Bread Wheat Genotypes Across Environments.” Turkish Journal of Agriculture and Forestry 26, no. 5: 275–279.

[pei370192-bib-0048] Kebede, G. , W. Worku , F. Feyissa , and H. Jifar . 2023. “Genotype by Environment Interaction for Agro‐Morphological Traits and Herbage Nutritive Values and Fodder Yield Stability in Oat ( *Avena sativa* L.) Using AMMI Analysis in Ethiopia.” Journal of Agriculture and Food Research 14: 100862.

[pei370192-bib-0049] Kilic, H. 2014. “Additive Main Effects and Multiplicative Interactions (AMMI) Analysis of Grain Yield in Barley Genotypes Across Environments.” Journal of Agricultural Sciences 20, no. 4: 337–344.

[pei370192-bib-0050] Kindie, Y. , B. Tesso , and B. Amsalu . 2021. “AMMI and GGE Biplot Analysis of Genotype by Environment Interaction and Yield Stability in Early Maturing Cowpea [ *Vigna unguiculata* (L) Walp] Landraces in Ethiopia.” Plant‐Environment Interactions 3, no. 1: 1–9. 37283694 10.1002/pei3.10068 PMC10168031

[pei370192-bib-0051] Kochian, L. V. , M. A. Piñeros , J. Liu , and J. V. Magalhaes . 2015. “Plant Adaptation to Acid Soils: The Molecular Basis for Crop Aluminum Resistance.” Annual Review of Plant Biology 66, no. 1: 571–598. 10.1146/annurev-arplant-043014-114822 25621514

[pei370192-bib-0052] Kroonenberg, P. M. 1995. “Introduction to Biplots for G × E Tables.” Centre for Statistics. Research Report 51. The University of Queensland, Brisbane, Australia, 22 pp.

[pei370192-bib-0053] Legesse, H. , M. Hussein , W. Walelign , and T. Bunyamin . 2015. “Genotype by Environmentinteraction and Stability of Chickpea ( *Cicer arietinum* (L.)).” Journal of Science and Development 3, no. 1: 13–25.

[pei370192-bib-0054] Maniruzzaman, M. , M. Z. Islam , F. Begum , M. A. A. Khan , M. Amiruzzaman , and A. Hossain . 2019. “Evaluation of Yield Stability of Seven Barley ( *Hordeum vulgare* L.) Genotypes in Multiple Environments Using GGE Biplot and AMMI Model.” Open Agriculture 4, no. 1: 284–293.

[pei370192-bib-0055] Matlala, M. , H. Shimelis , and J. Mashilo . 2019. “Genotype‐By‐Environment Interaction of Grain Yield Among Candidate Dryland Wheat Genotypes.” South African Journal of Plant and Soil 36, no. 4: 299–306.

[pei370192-bib-0056] Melese, A. , and H. Gebrekidan . 2014. “Phosphorus Status, Adsorption Characteristics, Kinetics and Its Availability to Wheat Crop as Influenced by Applications of Amendments on acid soils of Farta District, Northwestern Highlands of Ethiopia.” Doctoral Dissertation, Haramaya University.

[pei370192-bib-0057] Mesdag, J. , and A. G. Balkema‐Boomstra . 1984. “Varietal Differences for Reaction to High Soil Acidity and to Trace Elements; a Survey of Research in The Netherlands.” Fertilizer Research 5, no. 2: 213–233.

[pei370192-bib-0058] Mesfin, A. 2007. Nature and Management of Acid Soils in Ethiopia, 99. Haramaya University Printing Press.

[pei370192-bib-0059] Mohammadı, M. , R. Karımızadeh , N. Sabaghnıa , and M. K. Shefazadeh . 2012. “Genotype Ã Environment Interaction and Yield Stability Analysis of New Improved Bread Wheat Genotypes.” Turkish Journal of Field Crops 17, no. 1: 67–73.

[pei370192-bib-0060] Moldovan, V. , M. Moldovan , and R. Kadar . 2000. “Item From Romania.” SCA Agricultural Research Station, Turda.

[pei370192-bib-0061] Movahedi, H. , K. Mostafavi , M. Shams , and A. R. Golparvar . 2020. “AMMI Analysis of Genotype × Environment Interaction on Grain Yield of Sesame ( *Sesamum indicum* L.) Genotypes in Iran.” Biotechnology & Biotechnological Equipment 34, no. 1: 1013–1018.

[pei370192-bib-0062] Mukherjee, A. K. , N. K. Mohapatra , L. K. Bose , N. N. Jambhulkar , and P. Nayak . 2013. “Additive Main Effects and Multiplicative Interaction (AMMI) Analysis of GxE Interactions in Rice‐Blast Pathosystem to Identify Stable Resistant Genotypes.” African Journal of Agricultural Research 8, no. 44: 5492–5507.

[pei370192-bib-0063] Mulatu, B. , and S. Grando . 2006. “Barley Research and Development in Ethiopia.” In Proceedings of the Second National Barley Research and Development Review Workshop, pp. 28–30.

[pei370192-bib-0065] Nadeem, N. M. , and N. Islam . 2007. “AMMI Analysis of Some Upland Cotton Genotypes for Yield Stability in Different Milieus.” World Journal of Agricultural Sciences 3: 39–44.

[pei370192-bib-0066] Nehe, A. , B. Akin , T. Sanal , et al. 2019. “Genotype × Environment Interaction and Genetic Gain for Grain Yield and Grain Quality Traits in Turkish Spring Wheat Released Between 1964 and 2010.” PLoS One 14, no. 7: e0219432. 31318895 10.1371/journal.pone.0219432 PMC6638857

[pei370192-bib-0067] Ngeve, J. M. , and J. C. Bouwkamp . 1993. “Comparison of Statistical Methods to Assess Yield Stability in Sweetpotato.”

[pei370192-bib-0068] Obata, T. , and A. R. Fernie . 2012. “The Use of Metabolomics to Dissect Plant Responses to Abiotic Stresses.” Cellular and Molecular Life Sciences 69: 3225–3243. 22885821 10.1007/s00018-012-1091-5 PMC3437017

[pei370192-bib-0069] Oliveira, E. J. D. , J. P. X. D. Freitas , and O. N. D. Jesus . 2014. “AMMI Analysis of the Adaptability and Yield Stability of Yellow Passion Fruit Varieties.” Scientia Agricola 71: 139–145.

[pei370192-bib-0070] Pacheco‐Gil, R. Á. , M. Vargas , G. Alvarado , F. Rodríguez , J. Crossa , and J. Burgueño . 2015. “GEA‐R (Genotype × Environment Analysis With R for Windows) Version 4.1.”

[pei370192-bib-0071] Paradelo, R. , I. Virto , and C. Chenu . 2015. “Net Effect of Liming on Soil Organic Carbon Stocks: A Review.” Agriculture, Ecosystems & Environment 202: 98–107.

[pei370192-bib-0072] Purchase, J. L. 1997. “Parametric Analysis to Describe Genotype × Environment Interaction and Yield Stability in Winter Wheat.” Doctoral Dissertation, University of the Free State.

[pei370192-bib-0073] Rapsomanikis, G. 2015. The Economic Lives of Smallholder Farmers. An Analysis Based on Household Surveys. Food and Agriculture Organization.

[pei370192-bib-0074] Rodriguez, M. , D. Rau , R. Papa , and G. Attene . 2008. “Genotype by Environment Interactions in Barley ( *Hordeum vulgare* L.): Different Responses of Landraces, Recombinant Inbred Lines and Varieties to Mediterranean Environment.” Euphytica 163: 231–247.

[pei370192-bib-0075] SAS (Statistical Analysis System) . 2012. SAS/STAT Guide for Personal Computers, Version 9.4 Editions. SAS Institute Inc.

[pei370192-bib-0077] Sharma, R. C. , A. I. Morgounov , H. J. Braun , et al. 2010. “Identifying High Yielding Stable Winter Wheat Genotypes for Irrigated Environments in Central and West Asia.” Euphytica 171, no. 1: 53–64.

[pei370192-bib-0078] Sisay, T. , and T. Balemi . 2014. “Screening of Barley Cultivars ( *Hordeum vulgare* ssp. Vulgare L.) for Acid Soil Tolerance Under Greenhouse Condition.” Ethiopian Journal of Applied Science and Technology 5, no. 1: 58–84.

[pei370192-bib-0079] Solonechnyi, P. , N. Vasko , A. Naumov , et al. 2015. “GGE Biplot Analysis of Genotype by Environment Interaction of Spring Barley Varieties.”

[pei370192-bib-0080] Tamene, L. , T. Amede , J. Kihara , D. Tibebe , and S. Schulz . 2017. “A Review of Soil Fertility Management and Crop Response to Fertilizer Application in Ethiopia: Towards Development of Site‐ and Context‐Specific Fertilizer Recommendation.”

[pei370192-bib-0081] Taye, B. 2007. “An Overview of Acid Soils Their Management in Ethiopia.” Paper Presented in the Third International Workshop on Water Management (Waterman) Project, September 19–21, 2007. Haromaya, Ethiopia.

[pei370192-bib-0082] Tena, E. , F. Goshu , H. Mohamad , M. Tesfa , D. Tesfaye , and A. Seife . 2019. “Genotype× Environment Interaction by AMMI and GGE‐Biplot Analysis for Sugar Yield in Three Crop Cycles of Sugarcane (*Saccharum officinirum* L.) Clones in Ethiopia.” Cogent Food & Agriculture 5, no. 1: 1651925.

[pei370192-bib-0083] Thillainathan, M. , and G. C. J. Fernandez . 2001. “SAS Applications for Tai's Stability Analysis and AMMI Model in Genotype× Environmental Interaction (GEI) Effects.” Journal of Heredity 92, no. 4: 367–371. 11535655 10.1093/jhered/92.4.367

[pei370192-bib-0084] Tolessa, T. T. , G. Keneni , T. Sefera , M. Jarso , and Y. Bekele . 2013. “Genotype× Environment Interaction and Performance Stability for Grain Yield in Field Pea ( *Pisum sativum* L.) Genotypes.” International Journal of Plant Breeding 7, no. 2: 116–123.

[pei370192-bib-0085] Verma, R. P. S. , A. S. Kharab , J. Singh , V. K. Vishnu Kumar , I. Sharma , and A. V. Ajay Verma . 2016. “AMMI Model to Analyse G× E for Dual Purpose Barley in Multi‐Environment Trials.” Agricultural Science Digest 36, no. 1: 9–16.

[pei370192-bib-0087] Wang, J. P. , R. Harsh , Z. Guo‐Ping , M. Neville , and M. X. Zhou . 2006. “Aluminium Tolerance in Barley ( *Hordeum vulgare* L.): Physiological Mechanisms, Genetics and Screening Methods.” Journal of Zhejiang University. Science. B 7: 769–787. 16972319 10.1631/jzus.2006.B0769 PMC1599801

[pei370192-bib-0088] Yan, W. 2001. “GGEbiplot‐A Windows Application for Graphical Analysis of Multienvironment Trial Data and Other Types of Two‐Way Data.” Agronomy Journal 93, no. 5: 1111–1118.

[pei370192-bib-0089] Yan, W. , and L. A. Hunt . 2001. “Interpretation of Genotype× Environment Interaction for Winter Wheat Yield in Ontario.” Crop Science 41, no. 1: 19–25.

[pei370192-bib-0091] Yan, W. , and M. S. Kang . 2002. GGE Biplot Analysis: A Graphical Tool for Breeders, Geneticists, and Agronomists. CRC press.

[pei370192-bib-0094] Yan, W. , and M. S. Kang . 2003. GGE Biplot Analysis: A Graphical Tool for Breeders, Geneticists, and Agronomists. University of Guelph.

[pei370192-bib-0092] Yan, W. , M. S. Kang , B. Ma , S. Woods , and P. L. Cornelius . 2007. “GGE Biplot vs. AMMI Analysis of Genotype‐By‐Environment Data.” Crop Science 47, no. 2: 643–653.

[pei370192-bib-0093] Yan, W. , and N. A. Tinker . 2005. “An Integrated Biplot Analysis System for Displaying, Interpreting, and Exploring Genotype× Environment Interaction.” Crop Science 45, no. 3: 1004–1016.

[pei370192-bib-0095] Yohane, E. N. , H. Shimelis , M. Laing , I. Mathew , and A. Shayanowako . 2021. “Genotype‐By‐Environment Interaction and Stability Analyses of Grain Yield in Pigeonpea [ *Cajanus cajan* (L.) Millspaugh].” Acta Agriculturae Scandinavica Section B Soil and Plant Science 71, no. 3: 145–155.

[pei370192-bib-0096] Zhao, Z. , J. F. Ma , K. Sato , and K. Takeda . 2003. “Differential Al Resistance and Citrate Secretion in Barley ( *Hordeum vulgare* L.).” Planta 217: 794–800. 12734756 10.1007/s00425-003-1043-2

[pei370192-bib-0097] Zobel, R. W. , M. J. Wright , and H. G. Gauch Jr. 1988. “Statistical Analysis of a Yield Trial.” Agronomy Journal 80, no. 3: 388–393.

